# Involvement of RBP4 in Diabetic Atherosclerosis and the Role of Vitamin D Intervention

**DOI:** 10.1155/2018/7329861

**Published:** 2018-08-16

**Authors:** Wan Zhou, Shan-Dong Ye, Chao Chen, Wei Wang

**Affiliations:** Department of Endocrinology, Anhui Provincial Hospital, The First Affiliated Hospital of USTC, Division of Life Science and Medicine, University of Science and Technology of China, Hefei 230001, China

## Abstract

The purposes of this study were to evaluate the expression of retinol-binding protein 4 (RBP4) in diabetic rats with atherosclerosis and to investigate the role of vitamin D intervention. Male Wistar rats were randomly divided into 4 groups, including the control group (NC), the diabetic rats (DM1), the untreated diabetic atherosclerosis rats (DM2), and the vitamin D-treated diabetic atherosclerosis rats (DM3). The levels of serum and adipose RBP4, fasting insulin (FINS), fasting plasma glucose (FPG), total cholesterol (TC), high-density lipoprotein cholesterol (HDL-c), triglycerides (TG), low-density lipoprotein cholesterol (LDL-c), 25-hydroxyvitamin D [25(OH)D], C-reactive protein (CRP), and systolic blood pressure (SBP) were measured. Homeostasis model assessment of insulin resistance (HOMA-IR), homeostasis model assessment *β*-cell function index (HOMA-*β*), and atherogenic indexes (AI) were calculated. Compared with group NC, the levels of RBP4, TG, LDL-c, FPG, FINS, CRP, AI1, AI2, SBP, and HOMA-IR increased, while the levels of HDL-c, 25(OH)D, and HOMA-*β* decreased in groups DM1 and DM2. After 8 weeks of vitamin D supplementation in group DM3, the levels of 25(OH)D and HOMA-*β* increased and the levels of LDL-c, TC, HOMA-IR, FINS, CRP, RBP4, AI1, AI2, and SBP decreased significantly when compared with group DM2 (*P* < 0.05); Pearson analysis showed that serum RBP4 was positively correlated with TG, FINS, HOMA-IR, SBP, CRP, and AI and negatively correlated with 25(OH)D. In addition, multivariable logistic regression analysis showed that serum RBP4, SBP, and HDL-c were predictors for the presence of diabetic atherosclerosis. These findings suggested that RBP4 could involve in the improvement of diabetic atherosclerosis; vitamin D had the ability to decrease the level of RBP4 and eventually played an important role in preventing atherosclerosis in diabetes.

## 1. Introduction

It is reported that the worldwide prevalence of diabetes keeps increasing and may lead to a high incidence of macrovascular complications, such as heart failure, ischemic stroke, and nonfatal myocardial infarction. Diabetic macrovascular disease is a heterogeneous disorder characterized by multifactorial etiology and various processes, it has become the main leading cause of death and disability in diabetes, and its underlying potential pathological basis is atherosclerosis [1]. Thus, it is important to investigate the prevention strategies to control the occurrence of type 2 diabetes mellitus (T2DM) and to explore the different mechanisms of the development and progression of macrovascular diseases.

Adipose tissue is mainly responsible for energy storage and has been found to secrete a variety of biologically active substances, such as resistin, adiponectin, and leptin. Retinol-binding protein 4 (RBP4) is a newly discovered adipokine secreted by adipocytes and hepatocytes, and it is a specific transport protein for serum retinol [2]. Multiple studies have found that the level of RBP4 was elevated in obesity, T2DM, and other insulin-resistant diseases, which has explained the underlying association of RBP4 and insulin resistance (IR) as well as diabetes [3–6]. Recently, a research conducted in patients with high risks of cardiovascular diseases has found that the level of RBP4 increased significantly [7]. Meanwhile, emerging evidences have linked elevated level of serum RBP4 to cardiovascular diseases, such as atherosclerosis, hypertension, and coronary artery diseases (CAD) [8, 9], on the basis of population-based studies. Therefore, we hypothesized that RBP4 may serve as an important mediator in the development of diabetic atherosclerosis. However, the underlying role of RBP4 was still uncertain and the relationship between RBP4 and cardiovascular diseases remained controversial. For example, one study demonstrated that there was no correlation between RBP4 and vascular complications [10]. Several studies even showed a protective role of RBP4 on vascular in the population with a high risk of cardiovascular diseases [11, 12]. Therefore, a critical review of those studies is needed. This study aimed to investigate the potential mechanisms that could be involved in the role of RBP4 on diabetic atherosclerosis.

Vitamin D or also known as the “sunshine vitamin” is a kind of secosteroid, and serum 25-hydroxyvitamin D [25(OH)D] is a biomarker of vitamin D status in human body. Recent studies have found that besides the role of regulating calcium-phosphorus metabolism and bone calcification, vitamin D also has multiple biological effects. Meanwhile, it has been found related to nonbone metabolic diseases like cancer, obesity, and diabetes [13–16]. As vitamin D deficiency has become a worldwide concern, more and more scholars have revealed the relationship between vitamin D deficiency and cardiovascular diseases [17, 18]. However, few researchers have investigated the effects of vitamin D supplementation on diabetic atherosclerosis and the relationship between 25(OH) D and RBP4. Therefore, the objectives of our research are to investigate the relationship between RBP4 and vitamin D and to explore the role of vitamin D intervention through the construction of streptozotocin-induced diabetic and diabetic atherosclerosis rat models.

## 2. Materials and Methods

### 2.1. Ethic Statements

This research was approved by the Ethics Committee of Anhui Medical University, Medical Institution Animal Care and Research Advisory Committee (Hefei, China). According to the guidelines of Animal Institute of Nutrition (AIN) of USA, the animals were kept in the area with a temperature of 23 ± 2°C, a relative humidity of 55 ± 5%, and a light cycle of 14/10 h.

### 2.2. Experimental Animals

Two-week-old male Wistar rats with the weight of 160–200 g were purchased from the experimental animal center of Anhui Medical University and were divided into 3 groups randomly after adaptive feeding, including the normal control (NC, *n* = 8), the diabetes group (DM1, *n* = 13), and the diabetic atherosclerosis group (DAM, *n* = 25). The rats in groups NC, DM1, and DAM were fed up with a normal diet, a high-fat diet (including 2.5% cholesterol, 1% sodium cholate, 20% sugar, 10% lard, and 66.5% basal diet), and a high-fat diet with different components (including 3% cholesterol, 0.5% sodium cholate, 0.2% propylthiouracil, 5% sugar, 10% lard, and 81.3% basal diet), respectively. In the 4th week, a dose of streptozotocin (STZ; 30 mg/kg, Sigma-Aldrich, St. Louis, MO, USA) was added for rats in groups DM1 and DAM through intraperitoneal injection, whereas an equivalent volume of citric acid buffer was added for the rats in group NC. After 72 h, blood samples of rat tail veins in the 3 groups were collected to evaluate the level of plasma glucose and fasting plasma glucose (FPG) > 7.8 mmol/L was chosen as the T2DM model. Totally, 10 and 23 rats were successfully modeled in groups DM1 and DAM, respectively. Then, rats in group DAM were subjected to thoracic artery balloon injury. After the 8-week feeding, we subdivided the rats in DAM into 2 groups: the untreated diabetic atherosclerosis group (DM2, *n* = 12) and the vitamin D-treated diabetic atherosclerosis group (DM3, *n* = 11). A dose of 0.025 *μ*g·kg^−1^·d^−1^ vitamin D [19] (Haisheng Pharmaceutical Co. Ltd., Taizhou, China) dissolved in peanut oil was orally taken for rats of group DM3, while sodium chloride was used for rats of groups NC, DM1, and DM2 daily. Systolic blood pressure (SBP) was measured with the tail-cuff method through an electro-sphygmomanometer (type, RBP-1; China-Japan Friendship Research Institute, Beijing, China).

### 2.3. Collection of Samples

In the 16th week, all the experimental rats were fasted for 12 hours and then anesthetized with somnopentyl (pentobarbital sodium solution with a concentration of 64.8 mg/ml) and finally received euthanasia by collecting total blood from the left ventricle. After that, the blood samples were collected, then centrifuged to separate the serum, and stored at −80°C for detecting the levels of relevant serum components. Meanwhile, the visceral adipose tissue and other organs were collected, weighed, washed in saline, immersed in liquid nitrogen, and immediately stored at −80°C until use. Automatic biochemical analyzer (HITACHI 7600-020, Hitachi Ltd., Tokyo, Japan) was applied to detect the levels of low-density lipoprotein cholesterol (LDL-c), high-density lipoprotein cholesterol (HDL-c), triglycerides (TG), and total cholesterol (TC). Additionally, the insulin radioimmunoassay kit (Atom Hi-Tech Co., Ltd., China) was performed for the level of fasting insulin (FINS). We then centrifuged the remaining samples at 3000*g* for 20 min to separate the supernatant and used an enzyme-linked immunosorbent assay (ELISA) kit (USCN Business, Co., Ltd., Wuhan, China) to evaluate the level of C-reactive protein (CRP), serum RBP4, and 25(OH)D. HOMA-IR (homeostasis model assessment insulin resistance index) and HOMA-*β* (homeostasis model assessment *β*-cell function index) were used for estimating IR and the function of *β*-cells, respectively, and they were calculated by HOMA-IR = FINS × FPG/22.5 and HOMA-*β* = 20 × FINS/FPG − 3.5. As predictors of atherosclerosis, atherogenic indexes (AI) including AI1 and AI2 were estimated by the formulas AI1 = TC − HDL-c/HDL-c and AI2 = LDL-c/HDL-c. Subsequently, we isolated the thoracic aorta and performed hematoxylin and eosin (HE) staining. Finally, the mRNA and the quantitative protein expressions of RBP4 in visceral adipose tissues were detected by reverse transcription polymerase chain reaction (RT-PCR) analysis (Takara Biotechnology Co., Ltd., Dalian, China) and Western blot analysis, respectively.

### 2.4. ELISA Assay

To detect the expression of serum RBP4, we performed ELISA assay according to the manufacturer's process. The first process was incubation in which the RBP in the plasma samples was bound to a polyclonal rabbit anti-RBP antibody fixed on a microtitration plate. We then washed the plates and added a peroxidase-conjugated anti-RBP antibody, which was incubated for a certain time and then being washed. We applied tetramethylbenzidine as a peroxidase substrate for detection and quantification and calculated a dose-response curve of absorbance unit (optical density at 450 nm) with spline fitting from the standard values, which was then used to measure the RBP4 concentration of the samples. The intra- and intervariation of the assay were 5% and 9.75%, respectively.

### 2.5. RT-PCR Assay

Total RNA of the visceral adipose tissues was extracted by TRIzol (Invitrogen; Thermo Fisher Scientific, Inc., Carlsbad, CA, USA) according to the manufacturer's instructions, and the concentration and purity of the extracted RNA were detected by the ratio of OD 260 and 280. The MMLV reverse transcriptase and 2 *μ*g RNA were used for the synthesis of complementary deoxyribonucleic acid (cDNA). Syber Green II was used to perform the PCR reaction in PerkinElmer PCR System 9700 (Applied Biosystems, Foster, CA, USA) with the following steps: initial denaturation at 94°C for 30 seconds, followed by 30 PCR cycles of 94°C for 30 seconds, 60°C for 45 seconds, and 72°C for 45 seconds. The oligonucleotide primers were listed as follows: 5′-GACAAGGCTCGTTTCTCTGG-3′ (sense) and 5′-AAAGGAGGCTACACCCCAGT-3′ (antisense) for RBP4 and 5′-CACGATGGAGGGGCCGGACTCATC-3′ (sense) and 5′-TAAAGACCTCTATGCCAACACAGT-3′ (antisense) for *β*-actin. The amplified products were used for electrophoresis with 1.5% agarose gel (BioSens 805; Bio-Tech Co., Ltd., China), and the corresponding bands were obtained for optical density analysis (Gel-Pro Analyzer image analysis software version 3.0; Media Cybernetics, Inc., Rockville, USA).

### 2.6. Western Blot Assay

The tissue samples were firstly added to the tissue lysate solution (including Tris-HCl, pH 7.14, 150 mmol/L NaCl, 1 mmol/L EDTA, 1% Triton, 0.1% SDS, 5 mg/mL leupeptin, and 1 mmol/L PMSF) at a ratio of 1 : 20, and then, the lysates were centrifuged at 14000 ×g for 30 min at 4°C. BCA Protein Assay Reagent Kit (Thermo Fisher Scientific Inc., USA) was used to measure the protein concentrations of the samples, and sodium dodecyl sulfate-polyacrylamide gel was applied for protein electrophoresis. Then, we transfer the protein to a polyvinylidene fluoride (PVDF, Beyotime Biotechnology, Jiangsu, China) membrane, incubate the membrane in TPBS (phosphate-buffered saline containing 0.05% Tween 20) with 5% non-fat milk at room temperature for 2 h to block nonspecific binding, and use the anti-RBP4 antibody as the primary antibody at a dilution of 1 : 3000. After washing three times with TPBS, the PVDF membrane was then incubated with a horseradish peroxidase-conjugated secondary antibody at a dilution of 1 : 4000 at room temperature for 2 h. Finally, the Gel-Pro Analyzer software was used to analyze the bands captured on X-ray films.

### 2.7. Statistical Analysis

SPSS 19.0 (Statistical Package for the Social Science, SPSS Inc., Chicago, IL) was used for all the statistical analysis. All the experimental data were represented as mean ± standard deviation. Student's *t*-test or one-way analysis of variance (ANOVA) was performed for differences between different groups. Pearson correlation analysis was used for correlation between different variables. Multivariable logistic regression analysis was performed to measure the ORs and 95% confidence intervals of diabetic atherosclerosis. All *P* values were two-tailed, and *P* < 0.05 was considered statistically significant.

## 3. Results

### 3.1. HE Staining of Thoracic Aortas

**Figure 1:**
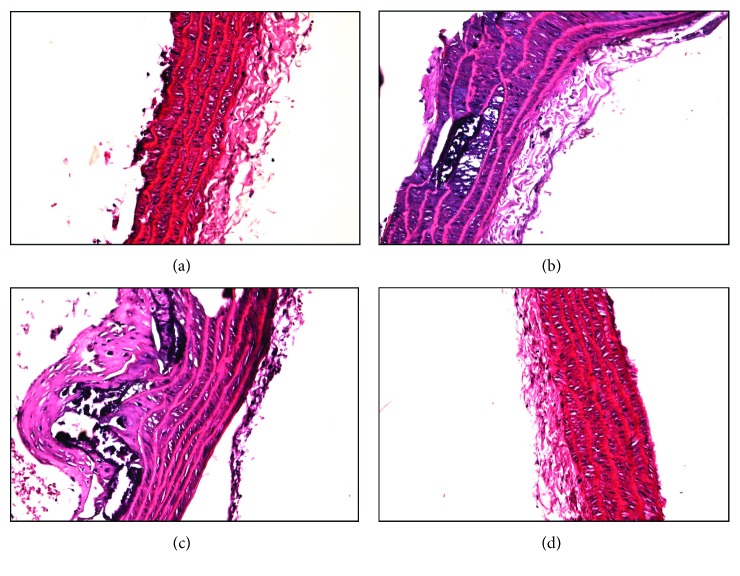
Thoracic aortas (hematoxylin and eosin staining): (a) normal control, (a) DM1, (c) DM2, and (d) DM3 groups.

Results showed that in group NC (Figure 1(a)), the thickening of the aortic wall was not obvious and the membrane of endothelial cells was long and thin with clear layers; in group DM1 (Figure 1(b)), the vascular intima was rough and the endothelial cells were normally arranged; in group DM2 (Figure 1(c)), the vascular intima was thickened and proceeded into the lumen, the endothelial cells were arranged in disorder, the subendocardial elastic tissue was ruptured, and multiple foam cells and calcification appeared; and in group DM3 (Figure 1(d)), the vascular intima was smooth, thin, and complete and the cells were arranged neatly. All the above results confirmed the successful construction of the diabetic atherosclerosis model.

### 3.2. Biochemical Analysis

**Figure 2:**
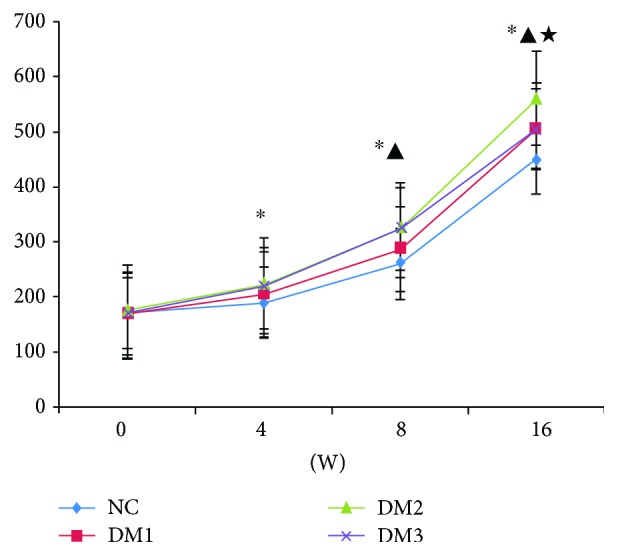
Comparison of weight in each group. ^∗^*P* < 0.05 versus NC group. ^▲^*P* < 0.05 versus DM1 group. ^★^*P* < 0.05 versus DM2 group.

The weights of rats in groups DM1 and DM2 were greater than those in group NC at the 4th, 8th, and 16th weeks, and the increase was more significant in group DM2. After vitamin D supplementation, the weights of rats in group DM3 decreased compared to the ones in group DM2. The weight curves of all the rats are presented in Figure 2. As shown in Table 1, the levels of TG, LDL-c, FPG, FINS, SBP, CRP, AI1, AI2, and HOMA-IR increased, while the levels of HDL-c, 25(OH)D, and HOMA-*β* decreased in groups DM1 and DM2 compared to group NC (*P* < 0.05). Compared with group DM2, we found that the levels of 25(OH)D and HOMA-*β* increased significantly while the levels of LDL-c, TC, HOMA-IR, CRP, AI1, AI2, FINS, and SBP decreased in group DM3 after 8 weeks of treatment with vitamin D (*P* < 0.05).

### 3.3. Levels of Serum RBP4

As shown in Table 2, when compared with group NC, the concentration of serum RBP4 in groups DM1 and DM2 increased significantly, and the concentration of RBP4 in group DM2 was 1.4-fold greater than that in group DM1. After an 8-week treatment with vitamin D, the level of RBP4 decreased by 20% in group DM3 as compared with that in group DM2 (*P* < 0.05).

### 3.4. mRNA Expression of RBP4 in Adipose Tissue

**Figure 3:**
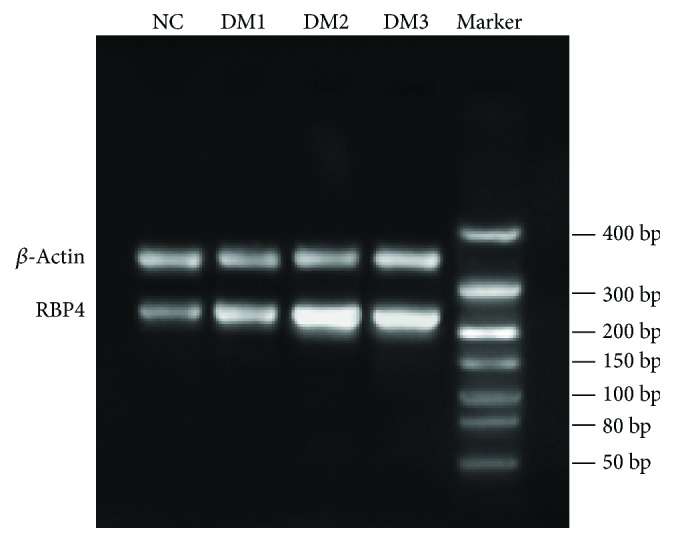
Comparison of adipose tissue RBP4 mRNA.

Results (shown in Figure 3) revealed that the mRNA expression of RBP4 in the adipose tissues was higher in groups DM1 (1.30 ± 0.24) and DM2 (2.16 ± 0.27) than in group NC (0.65 ± 0.18) and the increase of RBP4 expression was more significant in group DM2. Meanwhile, the expression was downregulated in group DM3 (1.75 ± 0.22) after vitamin D administration (*P* < 0.05).

### 3.5. Protein Expression of RBP4

**Figure 4:**
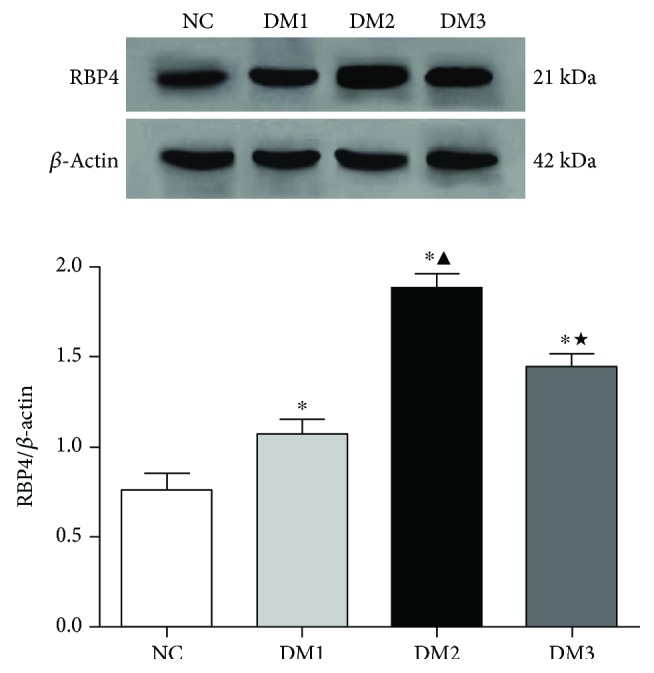
Comparison of RBP4 protein. ^∗^*P* < 0.05 versus NC group. ^▲^*P* < 0.05 versus DM1 group. ^★^*P* < 0.05 versus DM2 group.

The protein expressions of RBP4 were 0.75 ± 0.08, 1.1 ± 0.12, 1.8 ± 0.14, and 1.4 ± 0.10 in groups NC, DM1, DM2, and DM3, respectively. Additionally, the expression of RBP4 in group DM3 was markedly downregulated by vitamin D (*P* < 0.05) (shown in Figure 4).

### 3.6. Correlation among RBP4, 25(OH)D, and Other Indexes

Results of the Pearson correlation analysis revealed that the serum and adipose RBP4 were positively correlated with TG, FINS, CRP, AI1, AI2, SBP, and HOMA-IR and negatively correlated with 25(OH)D. However, the level of 25(OH)D was positively associated with HDL-c and HOMA-*β* and inversely correlated with RBP4, AI1, AI2, HOMA-IR, and LDL-c (shown in Table 2).

### 3.7. Independent Predictors for the Presence of Atherosclerosis

Multivariate logistic regression analysis is shown in Table 3, which was applied for all data obtained from diabetes rats. The occurrence of atherosclerosis was represented as a dependent variable, and the factors in groups DM1, DM2, and DM3 (shown in Table 1) were used as independent variables and included in the univariate logistic regression analysis. RBP4, SBP, and HDL-c were finally included in the regression model. Results of the multivariate logistic regression analysis revealed that RBP4 and SBP were risk factors for atherosclerosis and HDL-c might represent as a protective factor.

## 4. Discussion

Diabetes and atherosclerosis may exhibit common pathology, although the underlying mechanisms are still being explored. Systemic factors accompanied with diabetes, such as dyslipidaemia and hypertension, are thought to affect the development of diabetic vascular diseases. In addition, IR and hyperglycemia are the most two important factors [20]. In the present study, we observed that RBP4, SBP, and HDL-c were predictors for diabetic atherosclerosis through logistic regression.

RBP4 is a 21 kDa secreted protein elevated in insulin resistant states such as obesity and T2DM. In the present study, we found that the levels of adipose tissue and serum RBP4 in the DM groups especially in the diabetic atherosclerosis group were higher than those in the NC group. This phenomenon was in accordance with the previous results that RBP4 increased in diabetes patients with coronary heart disease and cerebral infarction and was negatively associated with carotid intima-media thickness [21–23]. The correlation between RBP4 and cardiovascular diseases was also found in studies conducted in a large number of elderly patients [24] or nonobese diabetic subjects [25], which indicated that the increased levels of RBP4 might be an independent risk factor and a diagnostic or prognostic marker for diabetes complicated with cardiovascular diseases. Results of our study suggested that RBP4 was associated with risk factors of cardiovascular disorders such as HOMA-IR, TG, SBP, CRP, AI1, and AI2 and was a predictor for diabetic atherosclerosis, which implied that RBP4 could involve in many pathways on the formation of atherosclerosis. The potential mechanisms are as follows. (1) In the present study, RBP4 had a strong correlation with HOMA-IR. Elevated RBP4 levels cause IR by inhibiting phosphatidylinositol 3 kinase (PI3K) activity in the skeletal muscle and increasing phosphoenolpyruvate carboxylase expression in the liver [2], and RBP4 might also cause IR through a retinol-dependent mechanism [26]. (2) The correlation between TG and RBP4 in our study may be represented as the relation among RBP4, obesity, and lipid disorder. Mohapatra et al. [27] has reported that RBP4 concentrations in T2DM complicated with cardiovascular diseases are associated with imbalances in lipid and glucose metabolism. Increased RBP4 could directly act to induce the expression of phosphoenolpyruvate carboxykinase, increase glucose production, and reduce insulin action to suppress glucose production in hepatocytes [2, 28]. RBP4 can also increase the expression of the gene encoding fatty acid syntheses of adipose tissues in a manner predominantly correlating with visceral fat accumulation [29]. (3) Obesity is identified as a low-grade chronic inflammation. Several studies observed that RBP4 increased in obesity and its complications including T2DM [30], metabolic syndrome [31], and cardiovascular diseases [32] as a chronic inflammatory state. There was a positive correlation between RBP4 and CRP in the study, which suggested a possible role of RBP4 in adipose inflammation. A number of studies have also investigated a positive correlation between RBP4 and inflammatory markers, including CRP, IL-6, and TNF-*α* [33–35], which is in accordance with our study. RBP's role in inducing inflammation through NADPH and NF-*κ*B pathways occurs independently of STRA6 and retinol [36].

The function of 25(OH)D on adipokines is a research hotpot currently. Several researches have shown that vitamin D is correlated with adiponectin, leptin, and other adipokines [37], but few studies have focused on analyzing the relationship between 25(OH)D and RBP4. Metheniti et al. [38] reported that the level of 25(OH)D was low in ultraobese young females and was significantly associated with RBP4 and neutrophil gelatinase-associated lipocalin (NGAL). In the present study, the results showed that 25(OH)D was negatively correlated with RBP4 and the RBP4 concentration decreased significantly after vitamin D supplementation. RBP4 appeared to have a mediating role in the association of 25(OH)D and multiple risk factors for cardiovascular diseases. One possible explanation was the direct role of 25(OH)D in modulating adipocyte signaling, which might result in an increased secretion of adiponectin [39]. Another explanation was that adipocytes extracted vitamin D (a molecule soluble in fat) from the circulation, stored it, and made it unavailable [40, 41]. Moreover, multiple researches also revealed that the lack of vitamin D could lead to greater adiposity by increasing the release of PTH, which might enhance calcium influx into adipocytes and eventually increase adipocytokines [42].

Multiple researches have confirmed the relationship between vitamin D deficiency and an increased risk for cardiovascular diseases in diabetes patients [18, 43, 44], which suggests that the vitamin D supplementation may play a potential protective role for the development of cardiovascular diseases. The clinic-based case-control study we conducted in 2013 showed that the incidence rate of lower extremity arterial disease in T2DM patients decreased after the intervention of vitamin D by improving IR and lipid metabolism and inhibiting inflammation [45]. As shown in this study, diabetic rats with atherosclerosis which received vitamin D therapy had lower LDL-c, TC, CRP, SBP, RBP4, HOMA-IR, AI1, and AI2 and higher HOMA-*β* and improved histological aspect of thoracic aorta compared with those without the treatment, which demonstrates that it is helpful to prevent atherosclerosis by controlling blood glucose, blood pressure, IR, and inflammation after vitamin D supplements. The following aspects might be important for the antiatherosclerotic effects of vitamin D. (1) As an endocrine suppressor of the renin angiotensin aldosterone system (RAAS), vitamin D can reduce the blood pressure by downregulating the RAAS [46]. (2) Vitamin D can reduce the risk of chronic inflammation by inhibiting the secretion of inflammatory markers [47]. Our results also showed that vitamin D treatment reduced the elevated levels of CRP, which suggests its anti-inflammatory role. (3) Hypovitaminosis D may have an adverse effect on endothelial function and eventually lead to an increased stiffness of the aorta [48]. (4) Vitamin D can improve insulin secretion by activating vitamin D receptors in pancreatic islet *β*-cells [49]. In the present study, 25(OH)D is associated with HOMA-IR, HOMA-*β*, and RBP4, and after vitamin D therapy, the three indexes were all improved. Vitamin D can also reduce peripheral IR by inhibiting the expression of peroxisome proliferator-activated receptor *γ* (PPAR-*γ*), which might partly contribute to the regulation of RBP4 [42]. (5) LDL-c is a recognized risk factor for atherosclerosis, but HDL-c has an antagonistic effect on atherosclerosis. Several studies have suggested that patients with vitamin D deficiency had higher LDL-c and lower HDL-c levels [50, 51]. Vitamin D supplementation could slow cholesterol deposition and further inhibit the foam cells formation in vascular wall by reducing the level of oxidized LDL-c.

## 5. Conclusion

The results revealed that RBP4 may contribute to the development of diabetes complicated with cardiovascular diseases through some potential mechanisms, such as insulin secretion and resistance, inflammatory reaction, dyslipidaemia, and lowering RBP4 levels, which may be an effective strategy for the prevention and treatment of T2DM macrovascular complications. Besides the bone metabolism role, vitamin D also has the ability to reduce the level of RBP4 and to provide vascular protection, which is partly associated with inhibiting cholesterol biosynthesis, relieving inflammation reaction, improving insulin resistance, and increasing insulin sensitivity. Detailed vitro experiments and longitudinal studies with larger sample size are still needed to confirm and extend these mechanisms in the future.

## Figures and Tables

**(a):** 

Group	*n*	TG (mmol/L)	LDL-c (mmol/L)	HDL-c (mmol/L)	TC (mmol/L)	FPG (mmol/L)
NC	8	0.57 ± 0.13	0.16 ± 0.03	0.71 ± 0.19	1.63 ± 0.32	5.12 ± 0.94
DM1	10	1.13 ± 0.37^∗^	0.31 ± 0.11^∗^	0.56 ± 0.12^∗^	1.69 ± 0.33	7.65 ± 1.42^∗^
DM2	11	2.16 ± 0.62^∗^^▲^	0.48 ± 0.15^∗^^▲^	0.49 ± 0.11^∗^	2.51 ± 0.43^∗^^▲^	10.05 ± 2.36^∗^^▲^
DM3	12	2.07 ± 0.59^∗^^▲^	0.34 ± 0.12^∗^^★^	0.52 ± 0.13^∗^	2.13 ± 0.39^∗^^▲★^	9.38 ± 2.13^∗^^▲^
*F*		22.914	11.943	4.528	12.029	12.606
*P*		<0.001	<0.001	0.008	<0.001	<0.001

^∗^ *P* < 0.05 versus NC group. ^▲^*P* < 0.05 versus DM1 group. ^★^*P* < 0.05 versus DM2 group. TG: triglycerides; LDL-c: low-density lipoprotein cholesterol; HDL-c: high-density lipoprotein cholesterol; TC: total cholesterol; FPG: fasting plasma glucose.

**(b):** 

Group	*n*	RBP4 (ng/mL)	HOMA-IR	HOMA-*β*	25(OH)D (nmol/L)	Weight
NC	8	13.58 ± 3.56	1.85 ± 0.36	80.25 ± 9.47	130.00 ± 10.34	430.00 ± 32.36
DM1	10	18.61 ± 4.89^∗^	3.97 ± 1.13^∗^	56.24 ± 7.61^∗^	108.00 ± 8.15^∗^	480.00 ± 35.63^∗^
DM2	11	25.34 ± 5.47^∗^^▲^	6.27 ± 2.56^∗^^▲^	42.84 ± 6.82^∗^^▲^	97.00 ± 7.72^∗^	530.00 ± 47.84^∗^^▲^
DM3	12	20.89 ± 4.76^∗^^★^	4.54 ± 1.21^∗^^★^	51.04 ± 7.73^∗^^★^	450.00 ± 38.59^∗^^▲★^	520.00 ± 45.19^∗^^▲^
*F*		9.721	12.121	37.487	664.27	10.97
*P*		<0.001	<0.001	<0.001	<0.001	<0.001

^∗^ *P* < 0.05 versus NC group. ^▲^*P* < 0.05 versus DM1 group. ^★^*P* < 0.05 versus DM2 group. RBP4: retinol-binding protein 4; HOMA-IR: homeostasis model assessment of insulin resistance; HOMA-*β*: homeostasis model assessment *β*-cell function index; 25(OH)D: 25-hydroxyvitamin D.

**(c):** 

Group	*n*	FINS (mU/L)	CRP (ng/mL)	AI1	AI2	SBP (mmHg)
NC	8	8.12 ± 1.54	339.8 ± 50.41	0.63 ± 0.14	0.23 ± 0.07	98.00 ± 8.38
DM1	10	11.67 ± 2.52^∗^	689.5 ± 71.13^∗^	0.69 ± 0.15	0.55 ± 0.12	105.00 ± 9.24
DM2	11	14.03 ± 3.12^∗^^▲^	1236.5 ± 103.16^∗^^▲^	1.51 ± 0.19^∗^^▲^	0.98 ± 0.36^∗^^▲^	123.00 ± 9.81^∗^^▲^
DM3	12	10.89 ± 2.27^∗^^★^	1004.7 ± 89.21^∗^^▲★^	1.13 ± 0.18^∗^^▲★^	0.65 ± 0.18^∗^^★^	114.00 ± 9.63^∗^^★^
*F*		8.988	204.501	59.400	18.308	12.946
*P*		<0.001	<0.001	<0.001	<0.001	<0.001

^∗^ *P* < 0.05 versus NC group. ^▲^*P* < 0.05 versus DM1 group. ^★^*P* < 0.05 versus DM2 group. FINS: fasting insulin; CRP: C-reactive protein; AI1: atherogenic index 1; AI2: atherogenic index 2; SBP: systolic blood pressure.

**Table 2:** Correlation among RBP4, 25(OH)D, and the other indicators in DM groups (*n* = 33).

Indicator	RBP4		25(OH)D	
	*r*	*P*	*r*	*P*
TG	0.304	0.036	−0.223	0.127
HDL-c	−0.282	0.077	0.382	0.007
LDL-c	0.195	0.229	−0.359	0.018
TC	0.136	0.157	0.093	0.407
FPG	0.079	0.595	−0.043	0.793
FINS	0.376	0.008	−0.129	0.429
Weight	0.096	0.556	−0.103	0.529
RBP4	—	—	−0.324	0.025
HOMA-IR	0.513	<0.001	−0.428	0.002
HOMA-*β*	−0.281	0.079	0.311	0.031
SBP	0.363	0.011	−0.270	0.092
CRP	0.342	0.018	0.172	0.138
AI1	0.402	<0.001	−0.314	0.035
AI2	0.419	<0.001	−0.378	0.002
25(OH)D	−0.328	0.023	—	—

TG: triglycerides; HDL-c: high-density lipoprotein cholesterol; LDL-c: low-density lipoprotein cholesterol; TC: total cholesterol; FPG: fasting plasma glucose; FINS: fasting insulin; RBP4: retinol-binding protein 4; HOMA-IR: homeostasis model assessment of insulin resistance; HOMA-*β*: homeostasis model assessment *β*-cell function index; SBP: systolic blood pressure; CRP: C-reactive protein; AI1: atherogenic index; AI2: atherogenic index 2; 25(OH)D: 25-hydroxyvitamin D.

**Table 3:** Multivariable logistic regression in DM groups (*n* = 33).

Variable	*B*	SE	Wald	OR	95% CI	*P*
RBP4	0.652	0.308	4.486	1.920	1.344–3.594	0.019
HDL-c	−1.118	0.399	7.848	0.327	0.216–0.761	0.011
SBP	0.349	0.186	3.511	1.417	1.053–3.080	0.034
Constant	4.907	0.715	—	—	—	0.008

RBP4: retinol-binding protein 4; HDL-c: high-density lipoprotein cholesterol; SBP: systolic blood pressure; *B*: *β* value; SE: standard error; Wald: *χ*^2^; OR: odds ratio; CI: confidence interval; Sig: significance.
